# Identification of lineage-specific epigenetic regulators FOXA1 and GRHL2 through chromatin accessibility profiling in breast cancer cell lines

**DOI:** 10.1038/s41417-024-00745-z

**Published:** 2024-03-01

**Authors:** Liying Yang, Kohei Kumegawa, Sumito Saeki, Tomoyoshi Nakadai, Reo Maruyama

**Affiliations:** 1 Project for Cancer Epigenomics, Cancer Institute, Japanese Foundation for Cancer Research, Tokyo, Japan; 2 Cancer Cell Diversity Project, NEXT-Ganken Program, Japanese Foundation for Cancer Research, Tokyo, Japan; 3 Breast Surgical Oncology, Breast Oncology Center, Cancer Institute Hospital, Japanese Foundation for Cancer Research, Tokyo, Japan

**Keywords:** Breast cancer, Gene regulation

## Abstract

Breast cancer is a heterogeneous disease, and breast cancer cell lines are invaluable for studying this heterogeneity. However, the epigenetic diversity across these cell lines remains poorly understood. In this study, we performed genome-wide chromatin accessibility analysis on 23 breast cancer cell lines, including 2 estrogen receptor (ER)-positive/human epidermal growth factor receptor 2 (HER2)-negative (ER+/HER2−), 3 ER+/HER2+, 3 HER2+, and 15 triple-negative breast cancer (TNBC) lines. These cell lines were classified into three groups based on their chromatin accessibility: the receptor-positive group (Group-P), TNBC basal group (Group-B), and TNBC mesenchymal group (Group-M). Motif enrichment analysis revealed that only Group-P exhibited coenrichment of forkhead box A1 (FOXA1) and grainyhead-like 2 (GRHL2) motifs, whereas Group-B was characterized by the presence of the GRHL2 motif without FOXA1. Notably, Group-M did not show enrichment of either FOXA1 or GRHL2 motifs. Furthermore, gene ontology analysis suggested that group-specific accessible regions were associated with their unique lineage characteristics. To investigate the epigenetic landscape regulatory roles of FOXA1 and GRHL2, we performed knockdown experiments targeting *FOXA1* and *GRHL2*, followed by assay for transposase-accessible chromatin sequencing analysis. The findings revealed that FOXA1 maintains Group-P–specific regions while suppressing Group-B–specific regions in Group-P cells. In contrast, GRHL2 preserves commonly accessible regions shared between Group-P and Group-B in Group-B cells, suggesting that FOXA1 and GRHL2 play a pivotal role in preserving distinct chromatin accessibility patterns for each group. Specifically, FOXA1 distinguishes between receptor-positive and TNBC cell lines, whereas GRHL2 distinguishes between basal-like and mesenchymal subtypes in TNBC lines.

## Introduction

Breast cancer is a complex disease that exhibits substantial heterogeneity. Current clinical practice relies on classifications based on gene expression patterns, such as intrinsic molecular subtypes and hormone receptor/human epidermal growth factor receptor 2 (HER2) expression [1–4]. However, previous studies have highlighted heterogeneity extending beyond gene expression across breast tumors [5, 6]. For example, we previously showed that a subset of estrogen receptor-positive (ER+) breast cancers displays reduced accessibility to ER-responsive elements, potentially leading to poor outcomes [7]. These findings underscore the critical need to identify epigenetic states for a more comprehensive understanding of breast cancer heterogeneity.

Breast cancer cell lines serve as pivotal models for studying breast cancer. They are typically categorized into various subtypes, primarily based on intrinsic subtype and/or receptor status. Lehmann et al. classified triple-negative breast cancer (TNBC) cell lines into six distinct groups: basal-like 1 (BL1), basal-like 2 (BL2), immunomodulatory, mesenchymal (M), mesenchymal stem-like (MSL), and luminal androgen receptor [8]. Multiple studies have highlighted transcriptional heterogeneity in breast cancer cell lines and primary tumors [9–11]. However, the epigenetic heterogeneity underlying the transcriptomes of these cell lines remains predominantly unexplored. Gaining insight into the epigenetic landscape of breast cancer cell lines is crucial for more precise disease modeling and elucidating key regulatory mechanisms.

We investigated the epigenetic heterogeneity of 23 breast cancer cell lines by profiling chromatin accessibility using the assay for transposase-accessible chromatin sequencing (ATAC-seq). We found that these cell lines could be categorized into three distinct groups based on chromatin accessibility patterns. Furthermore, we observed a significant correlation between these epigenetic groups and the motif enrichment of two transcription factors (TFs): forkhead box A1 (FOXA1) and grainyhead-like 2 (GRHL2). FOXA1 functions as a pioneer factor for ER, promoting luminal-lineage proliferation [12, 13], whereas GRHL2 is known to be involved in reprogramming ER signaling in breast cancer development [14]. Our knockdown experiments demonstrated that these TFs maintain accessibility to region-specific accessible regions. Consequently, we not only elucidated the epigenetic differences among breast cancer cell lines but also revealed a novel function of GRHL2 in distinguishing basal-like and mesenchymal characteristics in these cells.

## Results

### Classification of breast cancer cell lines into three distinct subgroups based on chromatin accessibility patterns

**Fig. 1:**
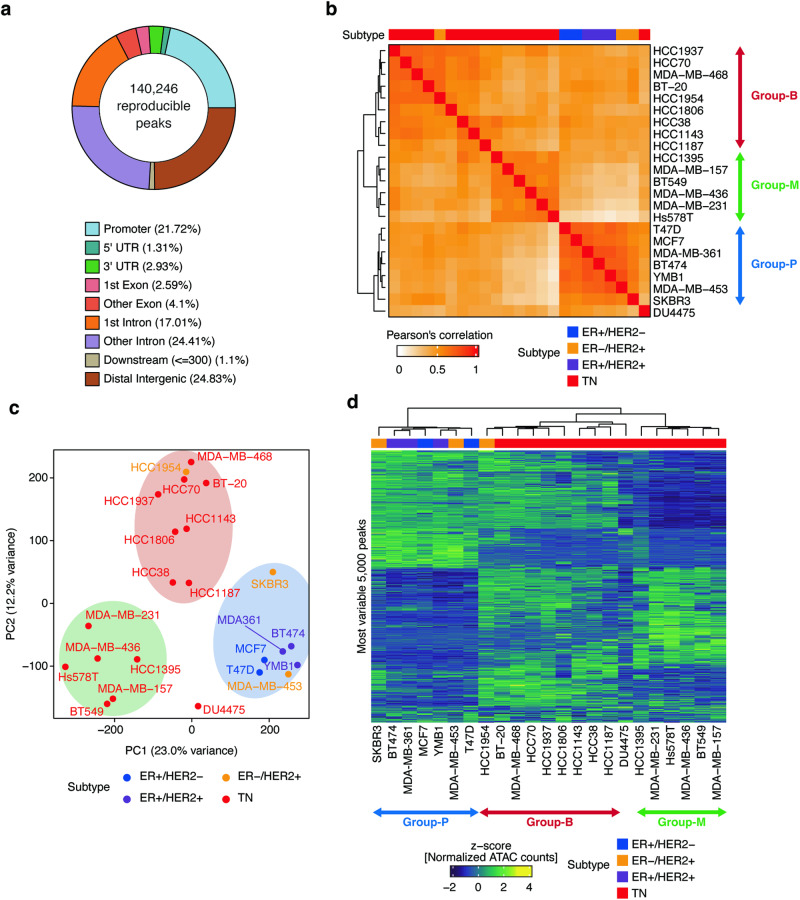
Classification of breast cancer cell lines based on chromatin accessibility profiles. **a** Genomic features of 140,246 merged reproducible peak sets. UTR: untranslated region. **b** Heatmap of Pearson’s correlations for ATAC-seq signals with all reproducible peaks. **c** Principal component analysis using the ATAC-seq profiles of cell lines. **d** Heatmap showing the chromatin accessibility of the top 5000 variable peaks. Annotations above the heatmap represent the receptor statuses of cell lines.

Using chromatin accessibility analysis, we investigated the epigenetic landscape in 23 breast cancer cell lines, including 2 ER+/HER2−, 3 ER+/HER2+, 3 HER2+, and 15 TNBC lines (Supplementary Table 1). All cell lines exceeded a predefined threshold for the transcription start site (TSS) enrichment score (≥5) and exhibited a unique fragmentation size distribution pattern with nucleosomal periodicity (Supplementary Fig. 1). Through peak calling, we identified 140,246 reproducible cis-regulatory elements (CREs) (Fig. 1a). A large proportion of these CREs were located in distal regions, including distal intergenic and intronic regions, whereas promoter elements constituted only 21.7% of the total CREs, consistent with previous findings [6, 7].

Based on CRE accessibility, correlation analysis among cell lines enabled the identification of three distinct groups: the receptor-positive group (Group-P), encompassing ER+ and/or HER2+ lines, including T47D, MCF7, MDA-MB-361, BT474, YMB1, MDA-MB-453, and SKBR3; the basal group (Group-B), primarily comprising basal-like TNBC lines, such as HCC1937 (BL1), MDA-MB-468 (BL1), HCC1806 (BL2), HCC38 (BL1), HCC1143 (BL1), and HCC70 (BL2) [8]; and the mesenchymal group (Group-M), including both mesenchymal and mesenchymal stem-like lines, such as BT549 (M), MDA-MB-436 (MSL), MDA-MB-231 (MSL), Hs578T (MSL), and MDA-MB-157 (MSL) [8] (Fig. 1b). Principal component analysis confirmed the segregation of these chromatin accessibility groupings (Fig. 1c). The cell line DU4475 displayed a unique chromatin accessibility pattern, likely due to distinct biological properties arising from its cutaneous metastatic nodule origin [15]. Notably, the accessibility pattern of the top 5000 most variable CREs indicated that Group-B exhibited high accessibility for CREs specific to both Group-P and Group-M (Fig. 1d). This suggests that Group-B has intermediate features of chromatin accessibility compared with the other groups. In total, 23 breast cancer cell lines were categorized into the three aforementioned groups based on chromatin accessibility.

### Motif enrichment of FOXA1 and GRHL2 associated with chromatin accessibility–based groups of breast cancer cell lines

**Fig. 2:**
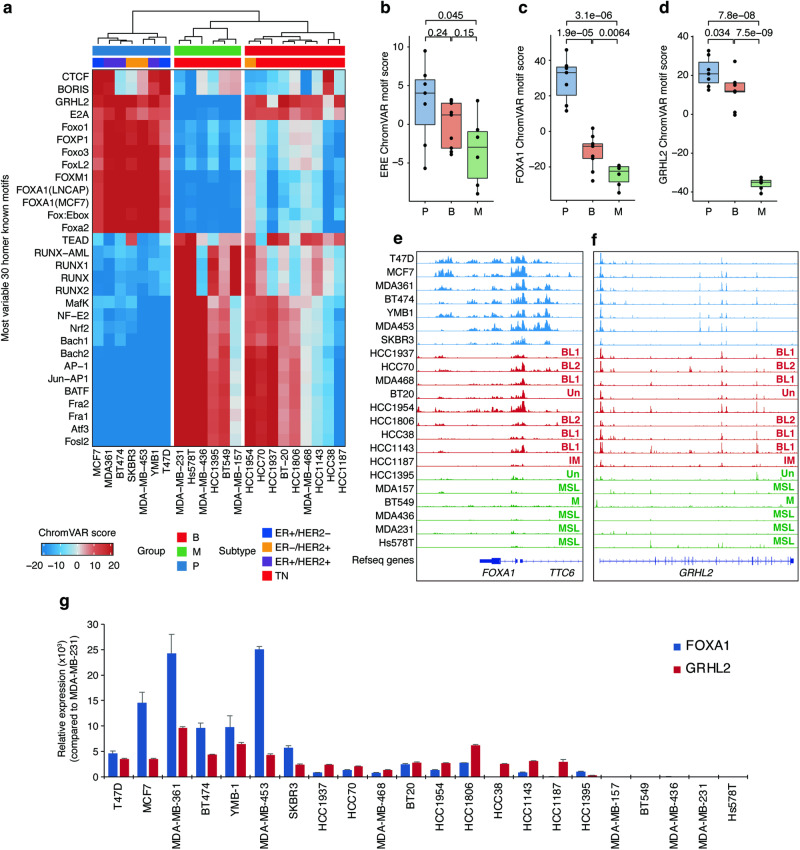
Enrichment of FOXA1 and GRHL2 binding motifs and chromatin accessibility in their coding regions. **a** Heatmap representing ChromVAR motif scores for the top 30 most variable motifs. Annotations above the heatmap indicate chromatin accessibility groups and the receptor statuses of cell lines. Boxplots showing motif scores across chromatin accessibility groups for ER (**b**), FOXA1 (**c**), and GRHL2 (**d**). *P*-values, calculated via Student’s t-test, are shown. Genome track view of the ATAC-seq profiles of cell lines at loci around *FOXA1* (**e**) and *GRHL2* (**f**). MSL mesenchymal stem-like, M mesenchymal, Un unclassified, IM immunomodulatory, BL1 basal-like1, BL2 basal-like2. **g** Bar plots showing the relative expression of FOXA1 (blue) and GRHL2 (red) compared to MDA-MB-231. Error bars represent standard deviation.

To gain insight into the three distinct subgroups, we analyzed enrichment of TF binding motifs within chromatin accessible regions across cell lines. Clear differences in motif enrichment patterns were observed between Group-P and Group-M (Fig. 2a). In particular, motifs belonging to the forkhead TF family, E2A, and GRHL2 were highly enriched in Group-P but less enriched in Group-M. In contrast, TEAD, RUNX, and AP-1 family motifs exhibited greater enrichment in Group-M, underscoring their crucial role in the mesenchymal state of breast cancer [16–18]. Notably, Group-B showed low enrichment of the forkhead motifs but displayed high enrichment of the GRHL2 motif (Fig. 2a).

We subsequently examined the luminal-lineage TFs ER and FOXA1. Enrichment of the ER-responsive element exhibited low variation among the three groups (Fig. 2b). In contrast, FOXA1 enrichment clearly distinguished Group-P from the other two groups (Fig. 2c), highlighting FOXA1’s importance as a TF associated with receptor-positive tumor lineages. Interestingly, GRHL2 motif enrichment remained consistently high in both Group-P and Group-B but was significantly lower in Group-M (Fig. 2d). These results suggest that the coexistence of FOXA1 and GRHL2 is unique to Group-P, whereas Group-B is characterized by the absence of FOXA1 and the presence of GRHL2. In contrast, Group-M is characterized by the absence of both FOXA1 and GRHL2.

To investigate the transcriptional activity of *FOXA1* and *GRHL2*, we evaluated the accessibility of genomic loci near these two genes and analyzed their expression. Group-P cell lines showed substantial chromatin accessibility not only around the *FOXA1* TSS but also in regions upstream and downstream of *FOXA1*, indicating the presence of enhancer elements (Fig. 2e). In contrast, Group-B cell lines exhibited moderate accessibility at the *FOXA1* TSS but lacked accessibility in the enhancer regions observed in Group-P (Fig. 2e). The *GRHL2* TSS exhibited high accessibility in Group-P and Group-B but lower accessibility in Group-M (Fig. 2f). Consistent with these observations, the mRNA expression levels of both *FOXA1* and *GRHL2* are high in Group-P cell lines (Fig. 2g). However, in Group-B cell lines, *FOXA1* expression is lower than in Group-P, while *GRHL2* expression remains at the same level as in Group-P lines. In Group-M, both genes show very low expression. These findings are consistent with the motif enrichment analysis and underscore the relationship between FOXA1 and GRHL2 activities and chromatin accessibility patterns in breast cancer cell lines.

### Functional annotation of group-specific CREs reveals the distinct epigenetic landscape associated with each group’s unique properties

**Fig. 3:**
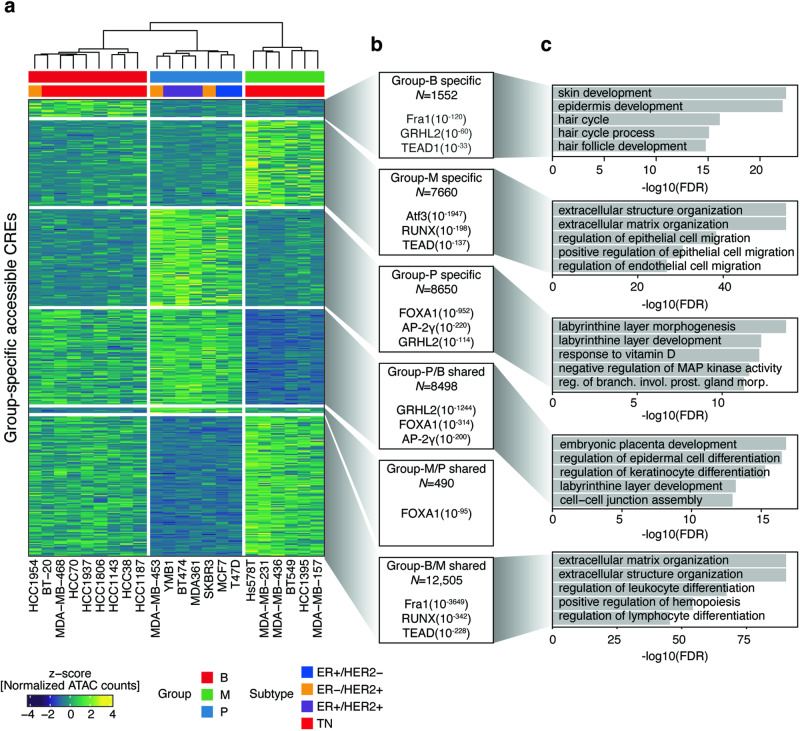
Group-specific or shared CREs. **a** Heatmap showing the chromatin accessibility of group-specific or shared CREs. Annotations above the heatmap indicate chromatin accessibility groups and the receptor statuses of cell lines. **b** Annotation of each CRE set and significantly enriched motifs. **c** Bar plots of GO enrichment obtained using GREAT analysis of each CRE set.

To explore epigenetic distinctions among groups, we conducted a differential accessibility analysis, resulting in the identification of six CRE sets: Group-P–specific (*N* = 8650), Group-B–specific (*N* = 1552), Group-M–specific (*N* = 7660), Group-P/B–shared (*N* = 8498), Group-B/M–shared (*N* = 12,505), and Group-M/P–shared (*N* = 490) (Fig. 3a**;** Supplementary Table 2). Consistent with prior findings indicating an intermediate epigenetic state in Group-B compared with the other groups (Fig. 1d), we observed a substantial number of accessible CREs shared between Group-P and Group-B, whereas the number of CREs shared between Group-P and Group-M was comparatively lower. Motif enrichment analysis revealed significant enrichment of FOXA1 and GRHL2 motifs in Group-P–specific and Group-P/B–shared CREs (Fig. 3b**;** Supplementary Table 3). Conversely, GRHL2 motifs were significantly enriched in Group-B–specific CREs, whereas FOXA1 motifs were absent.

We subsequently performed GREAT [19] Gene Ontology (GO) analyses for each set of CREs. We found that Group-P–specific CREs were associated with gland morphogenesis (Fig. 3c**;** Supplementary Table 4). Conversely, Group-M–specific CREs exhibited associations with processes related to extracellular matrix organization and epithelial cell migration, indicating their utility in characterizing the mesenchymal traits of Group-M cell lines. Notably, both Group-B–specific and Group-P/B–shared CREs showed associations with skin development, epidermal development, and keratinocyte differentiation. These findings suggest that group-specific CREs have a marked impact on establishing cellular identity. Collectively, these findings emphasize the critical role of FOXA1 in maintaining the epigenetic state of luminal or receptor-positive cells. Although both Group-B and Group-M are TNBC cell lines, they exhibit unique epigenetic profiles, with Group-B’s epigenetic state being notably influenced by GRHL2.

### FOXA1 and GRHL2 play a pivotal role in regulating the accessibility of Group-P–specific, Group-B–specific, and Group-P/B–shared CREs

Although motif enrichment analysis offers valuable insight into the potential activity of TFs, it cannot definitively establish their roles. Therefore, we conducted knockdown experiments targeting *FOXA1* (*FOXA1*-KD) and *GRHL2* (*GRHL2*-KD) in T47D (Group-P) and HCC38 (Group-B) to determine their involvement in the chromatin accessibility of Group-P–specific, Group-B–specific, and Group-P/B–shared CREs. We examined the expression analysis after knockdown, which showed the successful knockdown of both genes in these cell lines (Supplementary Fig. 2a–d).

**Fig. 4:**
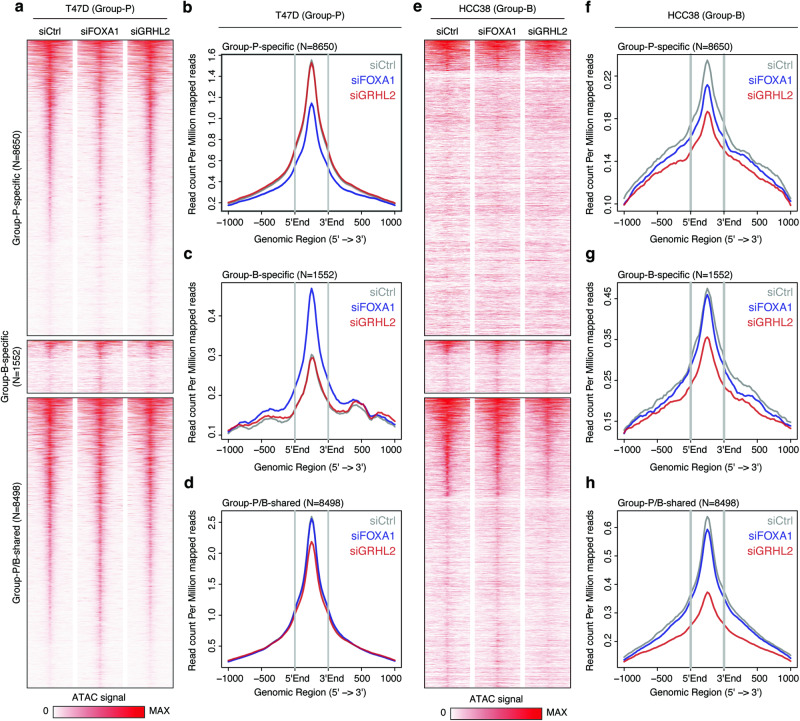
Effects of *FOXA1* or *GRHL2* knockdown on the chromatin accessibility of Group-P–specific, Group-B–specific, and Group-P/B–shared CREs. **a** Heatmap showing normalized ATAC-seq signals in Group-P–specific, Group-B**–**specific, and Group-P/B**–**shared CREs of control, *FOXA1* or *GRHL2* knockdown in T47D (Group-P) cells. Normalized read count profiles in Group-P**–**specific (**b**), Group-B**–**specific (**c**), and Group-P/B–shared (**d**) CREs of control, *FOXA1* or *GRHL2* knockdown in T47D (Group-P) cells. **e** Heatmap showing normalized ATAC-seq signals in Group-P–specific, Group-B**–**specific, and Group-P/B–shared CREs of control, *FOXA1* or *GRHL2* knockdown in HCC38 (Group-B) cells. Normalized read count profiles in Group-P–specific (**f**), Group-B**–**specific (**g**), and Group-P/B**–**shared (**h**) CREs of control, *FOXA1* or *GRHL2* knockdown in HCC38 (Group-B) cells.

We first knocked down *FOXA1* and *GRHL2* in T47D followed by ATAC-seq analysis. *FOXA1*-KD led to reduced accessibility to Group-P–specific CREs (Fig. 4a, b) and, surprisingly, increased accessibility to Group-B–specific CREs (Fig. 4c). In contrast, the accessibility of Group-P/B–shared CREs remained unaffected (Fig. 4d). *GRHL2*-KD caused a slight decrease in the accessibility of Group-P/B–shared CREs, with no effects on either Group-P–specific and Group-B–specific CREs (Fig. 4a–d). These findings suggest that *FOXA1* maintains the accessibility of Group-P–specific CREs while suppressing the accessibility of Group-B–specific CREs, with no significant effect on the accessibility of Group-P/B–shared CREs. Furthermore, GRHL2 is partially involved in regulating Group-P/B–shared CREs in the Group-P cell line.

Subsequently, *FOXA1*- and *GRHL2*-KD was conducted in HCC38, followed by ATAC-seq analysis. For Group-P–specific CREs, we observed low accessibility of these regions in HCC38 under three conditions, specifically a low number of reads per million mapped reads. (Fig. 4e, f). For Group-B–specific CREs, a slight decrease in accessibility was observed with *GRHL2*-KD, while *FOXA1*-KD showed no change (Fig. 4g). Furthermore, *GRHL2*-KD resulted in a reduction in Group-P/B–shared CRE accessibility, whereas *FOXA1*-KD did not produce the same effect (Fig. 4h). These results suggest that GRHL2 plays a critical role in preserving the shared epigenetic signature between Group-P and Group-B cell lines.

### *FOXA1*-KD reduces cell cycle in T47D cells, while *GRHL2*-KD activated a mesenchymal gene expression program in HCC38 cells

**Fig. 5:**
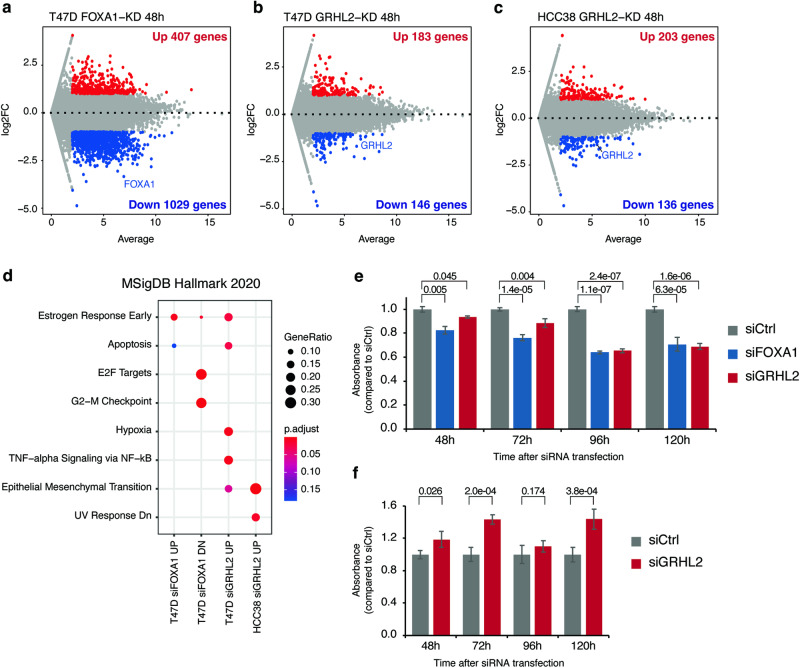
Transcription and cell proliferation changes of *FOXA1* or *GRHL2* knockdown. MA plots showing differentially expressed genes between the control and *FOXA1* knockdown in T47D (**a**), *GRHL2* knockdown in T47D (**b**), and *GRHL2* knockdown in HCC38 (**c**). **d** Gene enrichment analysis for each set of differentially expressed genes. No enriched terms were found for downregulated genes by siGRHL2 in T47D and in HCC38. Bar plots showing cell viability of T47D (**e**) and HCC38 (**f**) after siRNA transfection for 48, 72, 96, 120 h. Error bars represent standard deviations. *P*-values, calculated via Student’s *t* test, are shown.

We investigated the effects of chromatin accessibility changes, following *FOXA1* and *GRHL2* knockdown, on gene expression regulation and phenotypes. RNA-seq analysis revealed 407 upregulated and 1029 downregulated genes in T47D following *FOXA1*-KD, and 183 upregulated and 146 downregulated genes following *GRHL2*-KD (Fig. 5a, b; Supplementary Table 5). In HCC38, *GRHL2*-KD resulted in 203 upregulated and 136 downregulated genes (Fig. 5c; Supplementary Table 5). Gene enrichment analysis revealed that genes linked to the cell cycle were downregulated by *FOXA1*-KD in T47D (Fig. 5d), highlighting the critical role of FOXA1 in cell proliferation in luminal breast cancer [20]. In T47D cells, *GRHL2*-KD transcriptionally upregulated genes associated with apoptosis. Furthermore, genes upregulated by *GRHL2*-KD in both T47D and HCC38 were associated with epithelial-mesenchymal transition (EMT) (Fig. 5d). Notably, *GRHL2*-KD in HCC38 upregulated well-known EMT markers such as *VIM* and *VCAN*, as well as a key EMT regulator, *SNAI2* [21](Supplementary Table 5). These findings indicate that GRHL2 regulates gene expression, distinguishing between basal and mesenchymal lineages.

The cell proliferation assay showed that *FOXA1*-KD decreased cell proliferation in T47D cells, consistent with the decreased expression of cell cycle genes caused by *FOXA1*-KD (Fig. 5e). Similarly, *GRHL2*-KD resulted in decreased cell proliferation in T47D. In contrast, *GRHL2*-KD increased cell proliferation in HCC38 cells (Fig. 5f), suggesting a different role for GRHL2 in Group-P and Group-B cell lines.

Finally, we conducted survival analysis using The Cancer Genome Atlas invasive breast cancer (TCGA-BRCA) data. We found that elevated *FOXA1* expression may be associated with poorer prognosis in luminal A tumors (*P* = 0.067, Log-rank test; Supplementary Fig. 3a, b), but no such correlation was observed in basal tumors. Furthermore, elevated levels of *GRHL2* expression were significantly associated with a poorer prognosis in luminal A tumors (*P* = 0.038, Log-rank test; Supplementary Fig. 3c, d). However, this correlation was not statistically significant in basal tumors. Taken together, these findings suggest that FOXA1 plays a critical role in tumor proliferation in Group-P cells. Conversely, GRHL2 possesses a distinct function in maintaining cell viability in Group-P and sustaining the basal program in Group-B cells through the regulation of chromatin accessibility and gene expression.

## Discussion

Epigenetic regulation including non-coding RNAs [22, 23], chromatin organization [24], trans-acting transcription factors [25], and cis-acting regulatory elements [6], is a central machinery for cancer progression. In breast cancer, various TFs plays a pivotal role for promoting and/or suppressing cancer development and progression, highlighting that importance of better understanding of the functions of TFs and its targetable CREs to improve breast cancer diagnosis and treatment. In this study, we uncovered that well-known TFs, FOXA1 and GRHL2 are key epigenetic regulators distinguishing the diversity of genome-wide chromatin accessibility pattern across breast cancer cell lines.

FOXA1 is a well-established regulator, that acts as a pioneer factor for ER and drives estrogen-dependent proliferation in ER-positive breast cancer [12, 13]. Overexpression or activating mutations of FOXA1 promote breast cancer aggressiveness and are associated with poorer outcomes [26, 27]. Our study revealed that FOXA1 is highly expressed and showed increased accessibility to its binding motifs in Group-P including ER+ cell lines. Consistent with previous studies, *FOXA1*-KD resulted in the downregulation of genes associated with the cell cycle and decreased cell proliferation in an ER+/HER2– cell line. Furthermore, luminal tumors with high *FOXA1* expression tend to have a poorer prognosis. Interestingly, downregulation of FOXA1 not only reduced accessibility to Group-P–specific CREs, but also increased accessibility to Group-B–specific CREs. This finding emphasizes that FOXA1 has a pleiotropic role in ER+/HER2− breast cancer, promoting cell proliferation signaling and suppressing a distinct epigenetic program of basal-like tumors [28].

GRHL2 plays a crucial role in both normal developmental processes, particularly tubulogenesis, and cancer biology [29]. GRHL2 is a known tumor-promoting factor in breast cancer. It acts as a transcriptional regulator for genes associated with cell motility by influencing ER binding to chromatin [30]. Additionally, it serves as a reprogramming factor for ER binding sites during tumorigenesis [14] and collaborates with FOXA1 to establish endocrine resistance [31]. Our previous research has also shown that GRHL2 contributes to the epigenetic intratumor heterogeneity of ER+/HER2− breast cancer [32]. Collectively, these studies highlight the significant role of GRHL2 in the pathology of ER+/HER2− breast cancer. In contrast to these findings, our current study revealed that GRHL2 functions as an epigenetic regulator that distinguishes genome-wide chromatin accessibility between basal-like and mesenchymal types. While a previous study demonstrated that GRHL2 represses *ZEB1* expression and inhibits EMT in a basal-like cell line [33], our findings indicate that GRHL2 regulates not only the expression levels of individual genes but also maintains accessibility of numerous CREs associated with epithelial characteristics in multiple basal-like cell lines (Group-B lines). In the HCC38 cell line, which is basal-like, *GRHL2*-KD reduced the accessibility of CREs and increases the expression of EMT-related genes and cell proliferation. These findings indicate that GRHL2 affects not only genome-wide chromatin accessibility but also gene expression and cellular phenotype, contributing to basal-like characteristics. The study found that the EMT-associated factor upregulated by *GRHL2*-KD in HCC38 is *SNAI2*, not *ZEB1*, indicating that the function of GRHL2 in inhibiting EMT is context-dependent. The results suggest a heterogeneous mechanism for suppressing mesenchymal gene expression within basal-like cell lines, and the GRHL2-dependent CREs identified in this study could provide a common signature for regulating this process. Further exploration of these CREs may reveal a fundamental program for maintaining basal characteristics in basal-like breast cancer.

One question remaining in this study is which transcription factor regulates FOXA1 and GRHL2. Although previous research suggests that both FOXA1 and GRHL2 play a crucial role in the development and progression of breast cancer, there is limited understanding of their upstream regulators. The study identifies FOXA1 and GRHL2 as TFs associated with three distinct chromatin accessibility patterns across various cell lines. Similar enrichment patterns were observed with other FOX TFs and CTCF for FOXA1, and with E2A for GRHL2 (Fig. 2a). *CTCF*, known for its role as an insulator protein influencing chromatin organization [34], may regulate FOXA1 expression by modulating chromatin structure. The gene E2A has been linked to stemness, metastasis, and therapeutic resistance in breast cancer [35], however, there are no reports on its ability to distinguish between basal-like and mesenchymal types or its interaction with *GRHL2*. Therefore, it is important to explore the upstream regulators of FOXA1 and GRHL2 in future studies to gain a deeper understanding of their regulatory network in breast cancer.

In summary, our study provides insight into the epigenetic heterogeneity in breast cancer cell lines as well as the roles of FOXA1 and GRHL2 in shaping breast cancer properties. Although our data suggest that FOXA1 and GRHL2 contribute to maintaining the accessibility of group-specific CREs and distinct gene expression programs for each lineage, the precise mechanisms by which these factors influence phenotypes remain to be fully elucidated. Further extensive investigations are required to determine the nature of the interplay among these factors as well as their overall impact on breast cancer traits.

## Methods

### Cell culture

Breast cancer cell lines were purchased from the Japanese Collection of Research Bioresources, the American Type Culture Collection, or generously provided by Drs. Hitoshi Zembutsu and Yoshio Miki. Cells were cultured according to recommended guidelines, with specific details outlined in Supplementary Table 1.

### ATAC-seq experiment

ATAC-seq libraries were prepared following the Omni-ATAC protocol [36]. Briefly, 50,000 cells were lysed to release the nuclei and subjected to a transposition reaction using Tn5 transposase (Illumina). Transposed fragments underwent preamplification, quantification via real-time polymerase chain reaction, and subsequent amplification. Prepared libraries were sequenced on the Illumina MiSeq platform (Illumina) with paired-end reads (read 1, 75 bp; index 1, 8 bp; index 2, 8 bp, read 2, 75 bp).

### Knockdown experiment

T47D and HCC38 cells were transfected with small interfering RNA (siRNA) targeting FOXA1 (Ambion, s6687 and s6688) and GRHL2 (Ambion, s36754 and s36755) or with negative control siRNAs (Ambion, Negative Control #1 & Negative Control #2). All transfections were performed using Lipofectamine RNAiMAX Transfection Reagent (Invitrogen) following the manufacturer’s instructions. Cells were harvested 48 h post-transfection and then subjected to RT-qPCR, Western blot, RNA-seq and ATAC-seq analysis.

### Quantitative PCR

The QIAGEN RNeasy Plus Mini Kit was used to extract total RNA from the cells. The resulting RNA, with a quantity of 500 ng, was then used to create cDNA using the PrimeScript RT Master Mix Perfect Realtime (Takara), which was later diluted to a volume of 200 µL. Reverse transcription real-time polymerase chain reaction (RT–qPCR) was conducted using 2 µL of this cDNA for each reaction, employing TB Greeen^®^ Premix Ex Taq^TM^ II (Tli RNaseH Plus) on a StepOnePlus^TM^ Real-Time PCR System (Applied Biosystems). The relative expression of the *FOXA1* and *GRHL2* genes were measured using the delta-delta Ct method. For this, RNA from MBA-MB-231 was used as a reference, and the *ACTB* gene served as an internal control. The primers used in this study included FOXA1-F (GTGGCTCCAGGATGTTAGGA), FOXA1-R (CATGTTGCTGACCGGGAC), GRHL2-F (TGTTGAAGTCTCCCACAGTGA), GRHL2-R (AGTAGTGCTCGATGATGTTGTC), ACTB-F (GCCAACCGCGAGAAGATGA), and ACTB-R (AGCACAGCCTGGATAGCAAC).

### Western blot

Fifteen micrograms of protein were separated using SuperSep Ace, 10%, 17-well gels (Fujifilm) by electrophoresis. Following separation, proteins were transferred to PVDF membranes, which were then incubated overnight at 4 °C with the primary antibodies: anti-FOXA1 (Abcam, #ab23738) at a 1:1000 dilution and anti-GAPDH (HyTest, #5G4) at a 1:2000 dilution. After the primary antibody incubation, the membranes were washed and then incubated with horseradish peroxidase-conjugated secondary antibodies for 1 h at room temperature: anti-rabbit IgG, HRP-linked antibody (CST, #7074) at a 1:2000 dilution and anti-mouse IgG, HRP-linked antibody (CST, #7076) at a 1:2000 dilution. The protein bands were visualized using an enhanced chemiluminescence detection system.

### RNA-seq experiment

RNA was extracted from T47D and HCC38 cells after 48 h of siRNA treatment, using a previously detailed method. For RNA sequencing, 450–700 ng of the RNA was used to prepare each library using the SMARTer^®^ Stranded Total RNA Sample Prep Kit - HI Mammalian (Takara, 634874), according to the provided guidelines. The gene expression libraries produced were then sequenced on an Illumina NextSeq 550 system, using paired-end reads (75 bp for read1, 8 bp for the index, and 75 bp for read2).

### Cell proliferation assay

T47D and HCC38 cells were seeded onto 96-well plates at a density of 4 × 10^3^ cells per well for T47D and 2 × 10^3^ cells per well for HCC38. After 24 h, siRNA was transfected into the cells using the same method and concentration as previously described. Cell Counting Kit-8 (Dojindo) reagent was added to the cells at 48, 72, 96, and 120 h, and absorbance was measured.

### Data analysis

For ATAC-seq data analysis, we used Skewer [37] to trim Illumina adapter sequences, FastQC [38] for quality control of the sequenced reads, and Bowtie2 [39] for read removal from chrM or repeat sequences and alignment to the human genome hg38. To filter out duplicate reads, we used the Picard MarkDuplicates tool (http://broadinstitute.github.io/picard/). We assessed the normalized insertion profiles and fragment lengths of each ATAC fragment and calculated the TSS enrichment score for quality assessment. Subsequently, we performed peak calling analysis using MACS2. To generate a counts matrix, we employed a method established previously [6, 7] (Supplementary Methods). ChIPseeker was used for peak annotation [40]. Differential peak analysis was performed using the glmQLFTest package in edgeR [41]. Log2 fold change (log2FC) and false discovery rate (FDR) values were calculated, after which differential regions were identified as those with an absolute log2FC > 1 and an FDR < 0.01. Motif enrichment analysis was conducted using ChromVAR [42] and HOMER [43]. To compare ATAC-seq signals in the knockdown experiment, we used ngsplot [44].

For RNA-seq data analysis, we first trimmed raw reads to eliminate adaptor sequences using Skewer (version 0.2.2). These trimmed reads were then aligned to the human genome (hg38) using STAR (version 2.7.8a). Following this, we counted the aligned reads with featureCounts (version 2.0.10). After calculating log2-transformed transcripts per million (TPM), MA plots were generated and identified genes with log2 fold change >1 (upregulated) or < −1 (downregulated) and average expression >2 as differentially expressed genes.

For patient prognosis analysis, the RNA-seq data for TCGA-BRCA was obtained as a SummarizedExperiment object using the R package TCGAbiolinks [45]. A series of functions were used for this purpose: ‘GDCquery’ with parameters set to project ‘TCGA-BRCA,’ data category ‘Transcriptome Profiling,’ data type ‘Gene Expression Quantification,’ and workflow type ‘STAR-Counts,’ followed by ‘GDCdownload()’ and ‘GDCprepare()’. Survival data analysis was performed using the ‘survfit()’ function from the survival package and the ‘ggsurvplot()’ function from the survminer package. Patients were stratified by their FOXA1 or GRHL2 expression levels, with the top 33% categorized as the high group and the bottom 33% as the low group.

### Statistical analysis

To calculate FDR for the identification of specific CREs, we used quasi-likelihood F-tests through edgeR’s glmQLFTest function, as described above. To calculate the *p*-value for the comparison of chromVAR motif scores and cell proliferation assays, we used two-tailed Student’s *t* test.

### Supplementary information

Supplementary Figures

Supplementary Tables

Supplementary Method

## Data Availability

ATAC-seq and RNA-seq data have been deposited at Gene Expression Omnibus (GSE254216 and GSE254218) and are publicly available.

## Code availability

R code for reproducing the results can be found at https://github.com/KoheiKumegawa/BRCA_celllines.

## References

[1] Morganti S, Marra A, Crimini E, D’Amico P, Zagami P, Curigliano G (2022). Refining risk stratification in HR-positive/HER2-negative early breast cancer: how to select patients for treatment escalation?. Breast Cancer Res Treat.

[2] van ’t Veer LJ, Dai H, van de Vijver MJ, He YD, Hart AAM, Mao M (2002). Gene expression profiling predicts clinical outcome of breast cancer. Nature.

[3] Russnes HG, Lingjærde OC, Børresen-Dale A-L, Caldas C (2017). Breast Cancer Molecular Stratification. Am J Pathol.

[4] Parker JS, Mullins M, Cheang MCU, Leung S, Voduc D, Vickery T (2009). Supervised Risk Predictor of Breast Cancer Based on Intrinsic Subtypes. J Clin Oncol.

[5] Turashvili G, Brogi E. Tumor Heterogeneity in Breast Cancer. Front Med. 2017;4:227. 10.3389/fmed.2017.00227 PMC5727049 29276709

[6] Corces MR, Granja JM, Shams S, Louie BH, Seoane JA, Zhou W (2018). The chromatin accessibility landscape of primary human cancers. Science.

[7] Kumegawa K, Saeki S, Takahashi Y, Yang L, Osako T, Nakadai T (2023). Chromatin profile-based identification of a novel ER-positive breast cancer subgroup with reduced ER-responsive element accessibility. Br J Cancer.

[8] Lehmann BD, Bauer JA, Chen X, Sanders ME, Chakravarthy AB, Shyr Y (2011). Identification of human triple-negative breast cancer subtypes and preclinical models for selection of targeted therapies. J Clin Investig.

[9] Dai X, Cheng H, Bai Z, Li J (2017). Breast Cancer Cell Line Classification and Its Relevance with Breast Tumor Subtyping. J Cancer.

[10] Gambardella G, Viscido G, Tumaini B, Isacchi A, Bosotti R, di Bernardo D (2022). A single-cell analysis of breast cancer cell lines to study tumour heterogeneity and drug response. Nat Commun.

[11] Neve RM, Chin K, Fridlyand J, Yeh J, Baehner FL, Fevr T (2006). A collection of breast cancer cell lines for the study of functionally distinct cancer subtypes. Cancer Cell.

[12] Hurtado A, Holmes KA, Ross-Innes CS, Schmidt D, Carroll JS (2011). FOXA1 is a key determinant of estrogen receptor function and endocrine response. Nat Genet.

[13] Carroll JS, Liu XS, Brodsky AS, Li W, Meyer CA, Szary AJ (2005). Chromosome-Wide Mapping of Estrogen Receptor Binding Reveals Long-Range Regulation Requiring the Forkhead Protein FoxA1. Cell.

[14] Chi D, Singhal H, Li L, Xiao T, Liu W, Pun M (2019). Estrogen receptor signaling is reprogrammed during breast tumorigenesis. Proc Natl Acad Sci.

[15] Langlois AJ, Holder WD, Iglehart JD, Nelson-Rees WA, Wells SA, Bolognesi DP (1979). Morphological and biochemical properties of a new human breast cancer cell line. Cancer Res.

[16] Feldker N, Ferrazzi F, Schuhwerk H, Widholz SA, Guenther K, Frisch I (2020). Genome‐wide cooperation of EMT transcription factor ZEB1 with YAP and AP‐1 in breast cancer. EMBO J.

[17] Hong D, Fritz AJ, Zaidi SK, van Wijnen AJ, Nickerson JA, Imbalzano AN (2018). Epithelial‐to‐mesenchymal transition and cancer stem cells contribute to breast cancer heterogeneity. J Cell Physiol.

[18] Zanconato F, Forcato M, Battilana G, Azzolin L, Quaranta E, Bodega B (2015). Genome-wide association between YAP/TAZ/TEAD and AP-1 at enhancers drives oncogenic growth. Nat Cell Biol.

[19] McLean CY, Bristor D, Hiller M, Clarke SL, Schaar BT, Lowe CB (2010). GREAT improves functional interpretation of cis-regulatory regions. Nat Biotechnol.

[20] Yamaguchi N, Ito E, Azuma S, Honma R, Yanagisawa Y, Nishikawa A (2008). FoxA1 as a lineage-specific oncogene in luminal type breast cancer. Biochem Biophys Res Commun.

[21] Lu W, Kang Y (2019). Epithelial-Mesenchymal Plasticity in Cancer Progression and Metastasis. Dev Cell.

[22] Liu SJ, Dang HX, Lim DA, Feng FY, Maher CA (2021). Long noncoding RNAs in cancer metastasis. Nat Rev Cancer.

[23] Zou R, Shi Z, Xiao S, Ke Y, Tang H, Wu T (2019). Co-expression analysis and ceRNA network reveal eight novel potential lncRNA biomarkers in hepatocellular carcinoma. PeerJ.

[24] Wang M, Sunkel BD, Ray WC, Stanton BZ (2022). Chromatin structure in cancer. BMC Mol Cell Biol.

[25] He K, Feng Y, An S, Liu F, Xiang G (2022). Integrative epigenomic profiling reveal AP-1 is a key regulator in intrahepatic cholangiocarcinoma. Genomics.

[26] Fu X, Pereira R, De Angelis C, Veeraraghavan J, Nanda S, Qin L (2019). FOXA1 upregulation promotes enhancer and transcriptional reprogramming in endocrine-resistant breast cancer. Proc. Natl Acad. Sci.

[27] Arruabarrena-Aristorena A, Maag JLV, Kittane S, Cai Y, Karthaus WR, Ladewig E (2020). FOXA1 Mutations Reveal Distinct Chromatin Profiles and Influence Therapeutic Response in Breast Cancer. Cancer Cell.

[28] Bernardo GM, Bebek G, Ginther CL, Sizemore ST, Lozada KL, Miedler JD (2013). FOXA1 represses the molecular phenotype of basal breast cancer cells. Oncogene.

[29] Reese RM, Harrison MM, Alarid ET (2019). Grainyhead-like Protein 2: The Emerging Role in Hormone-Dependent Cancers and Epigenetics. Endocrinology.

[30] Reese RM, Helzer KT, Allen KO, Zheng C, Solodin N, Alarid ET (2022). GRHL2 Enhances Phosphorylated Estrogen Receptor (ER) Chromatin Binding and Regulates ER-Mediated Transcriptional Activation and Repression. Mol Cell Biol.

[31] Cocce KJ, Jasper JS, Desautels TK, Everett L, Wardell S, Westerling T (2019). The Lineage Determining Factor GRHL2 Collaborates with FOXA1 to Establish a Targetable Pathway in Endocrine Therapy-Resistant Breast Cancer. Cell Rep.

[32] Kumegawa K, Takahashi Y, Saeki S, Yang L, Nakadai T, Osako T (2022). GRHL2 motif is associated with intratumor heterogeneity of cis-regulatory elements in luminal breast cancer. NPJ Breast Cancer.

[33] Cieply B, Riley P, Pifer PM, Widmeyer J, Addison JB, Ivanov AV (2012). Suppression of the Epithelial–Mesenchymal Transition by Grainyhead-like-2. Cancer Res.

[34] Debaugny RE, Skok JA (2020). CTCF and CTCFL in cancer. Curr Opin Genet Dev.

[35] López-Menéndez C, Vázquez-Naharro A, Santos V, Dubus P, Santamaría PG, Martínez-Ramírez Á (2021). E2A Modulates Stemness, Metastasis, and Therapeutic Resistance of Breast Cancer. Cancer Res.

[36] Corces MR, Trevino AE, Hamilton EG, Greenside PG, Sinnott-Armstrong NA, Vesuna S (2017). An improved ATAC-seq protocol reduces background and enables interrogation of frozen tissues. Nat Methods.

[37] Jiang H, Lei R, Ding S-W, Zhu S (2014). Skewer: a fast and accurate adapter trimmer for next-generation sequencing paired-end reads. BMC Bioinformatics.

[38] Andrews S. FastQC: A Quality Control Tool for High Throughput Sequence Data [Online]. Available online at: http://www.bioinformatics.babraham.ac.uk/projects/fastqc/.

[39] Langmead B, Salzberg SL (2012). Fast gapped-read alignment with Bowtie 2. Nat Methods.

[40] Yu G, Wang L-G, He Q-Y (2015). ChIPseeker: an R/Bioconductor package for ChIP peak annotation, comparison and visualization. Bioinformatics.

[41] Robinson MD, McCarthy DJ, Smyth GK (2010). edgeR: a Bioconductor package for differential expression analysis of digital gene expression data. Bioinformatics.

[42] Schep AN, Wu B, Buenrostro JD, Greenleaf WJ (2017). ChromVAR: Inferring transcription-factor-associated accessibility from single-cell epigenomic data. Nat Methods.

[43] Heinz S, Benner C, Spann N, Bertolino E, Lin YC, Laslo P (2010). Simple Combinations of Lineage-Determining Transcription Factors Prime cis-Regulatory Elements Required for Macrophage and B Cell Identities. Mol Cell.

[44] Shen L, Shao N, Liu X, Nestler E (2014). ngs.plot: Quick mining and visualization of next-generation sequencing data by integrating genomic databases. BMC Genomics.

[45] Colaprico A, Silva TC, Olsen C, Garofano L, Cava C, Garolini D (2016). TCGAbiolinks: an R/Bioconductor package for integrative analysis of TCGA data. Nucleic Acids Res.

