# Supplementary material for: Identification of lineage-specific epigenetic regulators FOXA1 and GRHL2 through chromatin accessibility profiling in breast cancer cell lines
Source: Cancer Gene Ther. 2024 Mar 1;31(5):736–45. doi: 10.1038/s41417-024-00745-z (PMC11101334; doi:10.1038/s41417-024-00745-z)
Supplement: Supplementary file 1 — Supplementary Figures [file 41417_2024_745_MOESM1_ESM.pdf]

Supplementary Figure 1

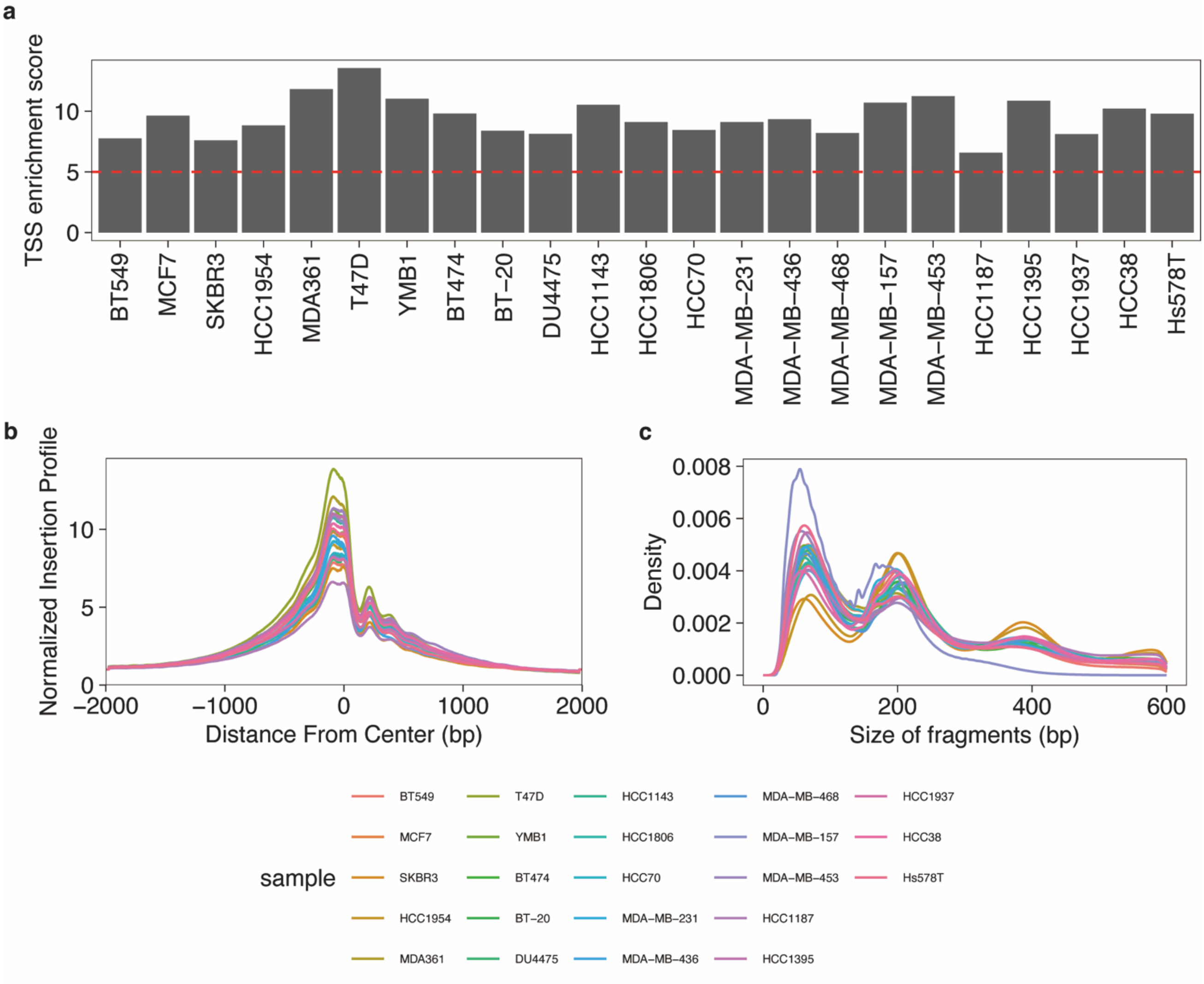

Supplementary Fig. 1. Quality assessment of ATAC-seq data. (a) Bar plot showing TSS enrichment scores of each cell line. (b) Normalized insertion profiles around TSSs. (c) Fragment size distribution.

Supplementary Figure 2

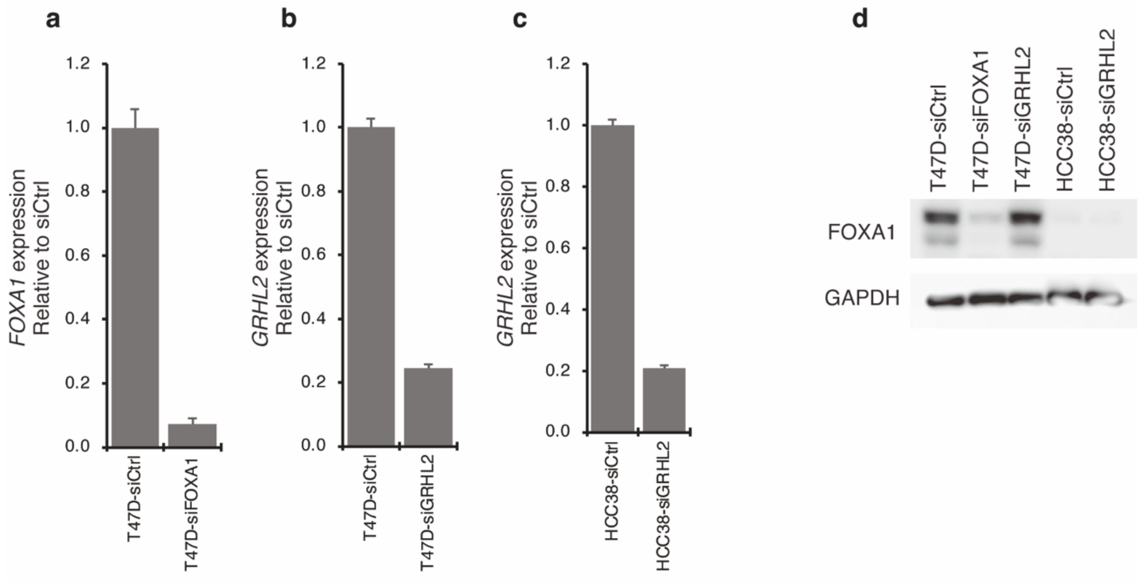

Supplementary Fig. 2. Knockdown efficiency. (a–c) Bar plot showing relative expression of *FOXA1* in T47D (a) and *GRHL2* knockdown in T47D (b) or HCC38 (c) compared to control, as determined by RT-qPCR. (d) Western blot showing FOXA1 protein levels for each condition.

Supplementary Figure 3

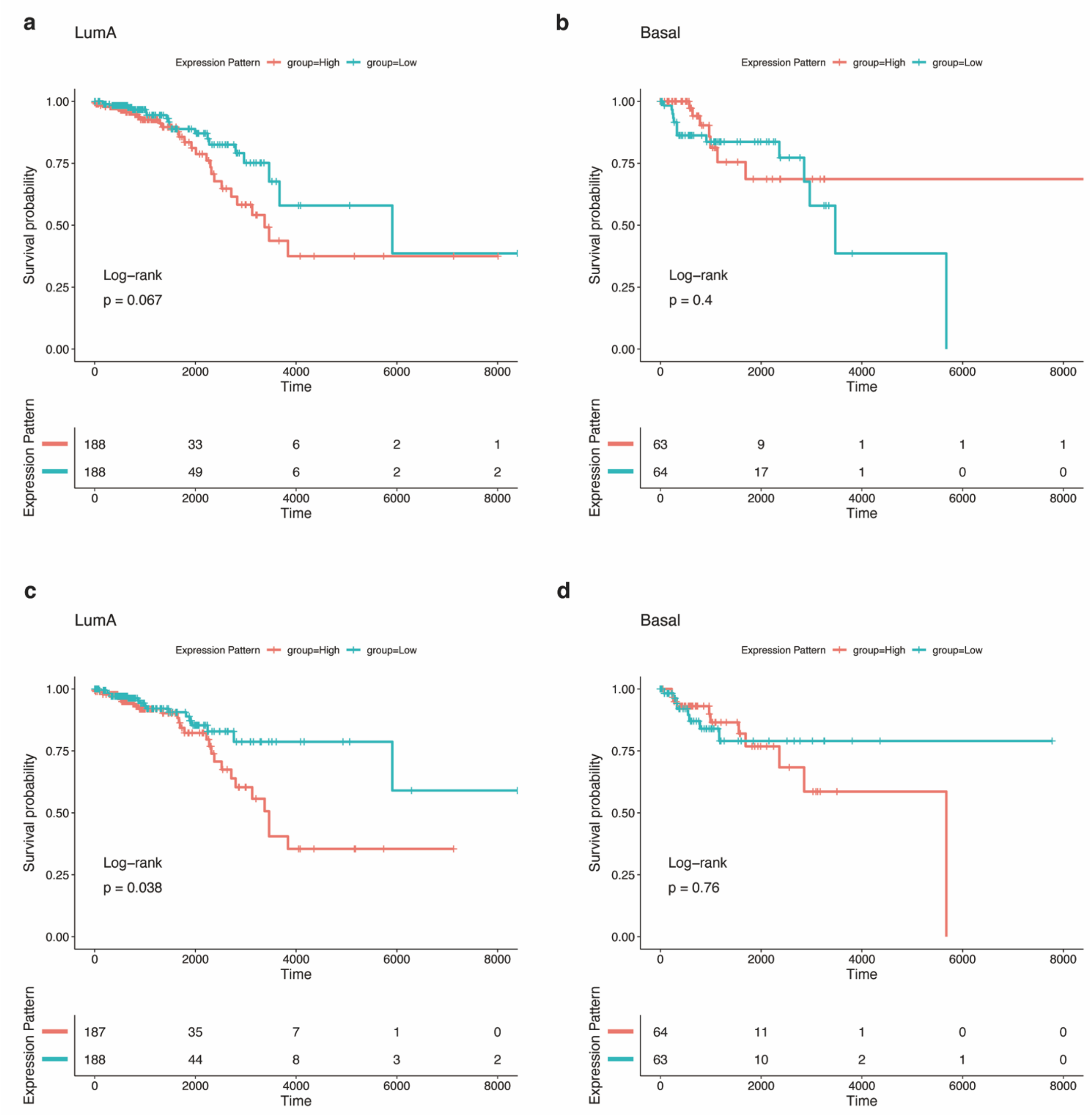

Supplementary Fig. 3. Survival analysis using TCGA-BRCA cohort. Kaplan-Meier plot of patients with high (top 33%) and low (bottom 33%) expression levels of *FOXA1* in luminal A cohort (a) and basal-like cohort (b), *GRHL2* in luminal A cohort (c) and basal-like cohort (d).
