# Supplementary material for: Identification of lineage-specific epigenetic regulators FOXA1 and GRHL2 through chromatin accessibility profiling in breast cancer cell lines
Source: Cancer Gene Ther. 2024 Mar 1;31(5):736–45. doi: 10.1038/s41417-024-00745-z (PMC11101334; doi:10.1038/s41417-024-00745-z)
Supplement: Supplementary file 3 — Supplementary Method [file 41417_2024_745_MOESM3_ESM.docx]

**Supplementary Methods**

Detailed method for data analysis

*ATAC-seq data analysis—processing and alignment*

The Fastq files were trimmed to remove Illumina Nextera adapter sequences using Skewer with the following options: “-f sanger -t 20 -m pe -x”. After trimming, sequencing quality validation was performed using FastQC. To remove reads originating from chrM or repeat sequences, pre-alignments were carried out to eliminate reads that might map to these regions using Bowtie2 with the options: “-k 1 -D 20 -R 3 -N 1 -L 20 -I S,1,0.50 -X 2000 --no-mixed --no-discordant”. Filtered reads were then aligned to the hg38 human reference genome using Bowtie2 with the options: “--very-sensitive -X 2000 -no-mixed --no-discordant”. To remove duplicate reads, the Picard MarkDuplicates tool (http://broadinstitute.github.io/picard/) was utilized with the options: “VALIDATION_STRINGENCY = LENIENT REMOVE_DUPLICATES = true”. The final aligned and de-duplicated BAM files were utilized in all subsequent analyses.

*ATAC-seq data analysis—quality check*

To assess the quality of each ATAC-seq profile, we employed two metrics: the enrichment of ATAC-seq accessibility at transcription start sites (TSSs) and the analysis of fragment length distribution. First, the BAM files were imported as Genomic Ranges objects in R using the “scanbam” command from Rsamtools. To correct for any offsets relative to the read start, we added 4 bp for the “+” stranded reads and subtracted 5 bp for the “−” stranded reads. For the TSS enrichment profiling, we extended each TSS position, which was obtained using the “transcripts(TxDb)” command from the “TxDb.Hsapiens.UCSC.hg38.knownGene” package, by 2000 bp in both directions. We then overlapped these extended regions with the insertions, representing either end of a fragment, using the “findOverlaps()” function. Subsequently, we calculated the distance between the insertions and the strand-corrected TSS. The number of insertions in each single-base bin was summed for further analysis. To normalize the values, we considered the accessibility at each position within ±1900–2000 bp from the TSS. The final TSS enrichment score was defined as the maximum enrichment value within ±50 bp of the TSS after smoothing using a rolling mean with a window size of 51 bp. For generating the fragment length distribution, we plotted the width of each fragment.

*ATAC-seq data analysis—peak calling and making a counts matrix*

To derive a high-quality peak set from a dataset comprising ATAC-seq profiles of 23 breast cancer cell lines, we adopted a comprehensive analysis approach. We initiated the process by conducting peak calling on the Tn5-corrected single-base insertions from each tumor sample. For this task, we employed MACS2, specifying the following parameters: “--shift -75 --extsize 150 --nomodel --call-summits --nolambda --keep-dup all -p 0.01”. Subsequently, we extended the summits of the identified peaks by 250 bp on both sides, ultimately achieving a fixed width of 501 bp for each peak. To enhance data quality, we filtered out regions designated in the ENCODE hg38 blacklist. Within each individual sample, we implemented an iterative procedure to remove overlapping peaks, retaining those with the most significant “score” values from the MACS2 output. This yielded a “sample peak set.” To further refine our analysis, we calculated the “Score per Million” for each peak by dividing its individual score by the sum of all peak scores within the respective sample, normalized to one million. Following this, we repeated the iterative removal procedure across the sample peak sets, this time based on the “Score per Million” values. The final step involved identifying a reproducible peak set, characterized by “Score per Million” values of ≥5 and overlapping peaks observed in at least two distinct samples. Peaks located on chromosome Y were excluded from consideration. This process culminated in a final set of high-quality peaks, each having a fixed width of 501 bp, extracted from the 23 ATAC-seq profiles. To determine the number of Tn5 insertions within each peak, we processed BAM files in R as Genomic Ranges objects using Rsamtools’ “scanbam()” function. Tn5 offsets were corrected by adding 4 bp for “+” stranded reads and subtracting 5 bp for “−” stranded reads. The corrected insertions were then counted using the “countOverlaps()” function. Subsequently, we normalized the resulting counts matrix using edgeR’s “cpm(log = TRUE, prior.count = 5)” and performed quantile normalization with preprocessCore’s “normalize.quantiles()“.

*ATAC-seq data analysis—profiling peaks and cell lines*

To annotate the peaks, we employed the “annotatePeak()” function from ChIPseeker with its default settings. Subsequently, we identified overlapping peaks between the JFCR–BRCA peaks and the TCGA–BRCA peaks using the “findOverlaps()” function. For calculating correlations between tumors, we used the “cor()” function with the “method” parameter set to ‘pearson’.

*Differential analysis – edgeR*

We utilized R package edgeR version 3.32.1 along with its glmQLFTest function to detect DARs within ATAC-seq data. The workflow encompassed the subsequent steps: Initially, we normalized library sizes to account for variations in sequencing depth across samples. This normalization was executed using the calcNormFactors(y, method = TMM) function. Following that, we estimated dispersion using the estimateDisp(y, design = design, robust = TRUE) function. After dispersion estimation, we conducted a generalized linear model (GLM) analysis using the glmQLFit(y, design = design) function. We computed log2 fold change (log2FC) and False Discovery Rate (FDR) values, then regions with an absolute log2FC exceeding 1 and an FDR below 0.01 were categorized as DARs.

*Motif enrichment analysis—ChromVAR and HOMER*

Motif enrichment scores were calculated using ChromVAR through the following steps: We introduced GC bias information into the analysis by applying the “addGCBias()” function. Then, we identified elements containing motifs of interest using the “matchMotifs()” function, leveraging motif annotation from the R package chromVARmotif’s “homer_pwms.” To establish a suitable background for comparative analysis, we obtained background peaks using the “getBackgroundPeaks()” function. Following this, motif deviations were computed using the “computeDeviations()” function. The resulting Z-scores of motif deviations, denoted as Motif scores, were then harnessed for subsequent analysis. Furthermore, for the motif enrichment analysis of the group-specific DARs, we employed HOMER v4.10's “findMotifsGenome.pl” tool, specifying the options “-size 200 -mask -nomotif.”
